# Reassessing the Evidence for Capacity Limits in Neural Signals Related to Working Memory

**DOI:** 10.1093/cercor/bhx351

**Published:** 2018-01-10

**Authors:** Paul M Bays

**Affiliations:** Department of Psychology, University of Cambridge, Downing St, Cambridge, UK

**Keywords:** electroencephalography, functional magnetic resonance imaging, resource model, short-term memory, slot model

## Abstract

In 2004, two landmark studies described the discovery of brain imaging (functional magnetic resonance imaging and electroencephalography) signals that increase with the number of items held in visual working memory (WM). These studies claimed that the signals leveled off (plateaued) once the number of memoranda reached the capacity of WM, as estimated by the prevailing model of the time. However, alternative models were not considered, and changing concepts of WM in the more than a decade since these studies were published necessitate a re-evaluation of their findings; newer models that provide the most accurate account of behavioral data do not incorporate a fixed limit on the number of items stored. Furthermore, an important claim made about the original studies, that signals plateau at each individual’s estimated capacity, has never been tested. Here, we pit the plateau model of signal strength against an alternative, saturation model, a biophysically plausible account in which signals increase continuously without plateau. We show that the saturation model provides a better description of the original data, challenging the assumption that imaging results provide evidence for a fixed item limit in WM.

One of the defining characteristics of working memory (WM) is its limited capacity, however, our understanding of the nature of this limitation has evolved (Ma, Husain, and Bays 2014). Traditionally it was assumed that there is a fixed upper limit on the number of items WM can hold at one time (Miller 1956; Luck and Vogel 1997), and methods were developed for estimating this number from performance on memory tasks such as change detection (Pashler 1988; Cowan 2001). More recently, it has been established that the fidelity with which information is stored in WM decreases monotonically with increasing number of items stored (Palmer 1990; Wilken and Ma 2004; Bays and Husain 2008). This finding is most parsimoniously explained by models in which WM is limited by a fixed quantity of a flexible representational resource, with no upper limit on the number of items stored. Resource models provide a consistently better description of behavioral performance than models based on a fixed item limit (Bays, Catalao, and Husain 2009; van den Berg et al. 2012; Keshvari, van den Berg, and Ma 2013; Bays 2014; van den Berg, Awh, and Ma 2014), even when those models incorporate resource-like characteristics (Zhang and Luck 2008).

Prior to the development of resource models, a number of studies sought neural signals consistent with the fixed capacities estimated from change detection. Todd and Marois (2004)looked in functional magnetic resonance imaging (fMRI) data for regions with activity consistent with an item limit (specifically, they used regressors weighted according to the behaviorally estimated number of items in memory). Their analysis identified a bilateral region of the intraparietal and intraoccipital sulci (IPS/IOS) in which activity initially increased with set size (the number of items in the memory array) and then appeared to plateau. Vogel and Machizawa (2004) observed similar behavior in a signal recorded at posterior electroencephalography (EEG) electrodes, the contralateral delay activity (CDA).

In each of these studies, the authors claimed to find evidence that signal strength reached a plateau at a set size corresponding approximately to the average capacity estimated from behavioral data (2.8 items in Vogel and Machizawa 2004; 3.4 items in Todd and Marois 2004). This conclusion was based on null hypothesis tests comparing signal strength at different set sizes: in Vogel and Machizawa (2004), *t*-tests revealed a significant difference between set sizes 2 and 4, but no significant change between 4 and 6; in the case of Todd & Marois (2004)a *t*-test comparing set sizes 4 and 8 was not significant, tests comparing lower set sizes were not reported.

These papers have been extensively cited as providing strong evidence for a fixed maximum number of items that can be stored in WM. A typical summary of Todd and Marois’ study is that “the signal in the intraparietal sulcus (IPS) during the delay period increases as the set size increases, reaching an asymptote at the individual subject’s VWM capacity” (Luck and Vogel 2013; see also Fukuda, Awh, and Vogel 2010). However, this interpretation can be questioned on several grounds.

First, the approach of identifying a plateau using a series of null hypothesis tests is statistically invalid, because a hypothesis test can never provide evidence in favour of the null (i.e., no change), only fail to reject it. Intuitively, consider that the conventional significance threshold of *P* < 0.05 is arbitrary; if the authors had used a different threshold, their method would have identified a plateau as occurring at a different set size; therefore, this approach is not a reliable method for identifying a plateau.

Second, contrary to what is often claimed, the imaging studies did not test whether the signal reached an asymptote (i.e., plateau) at each individual’s capacity. Indeed no attempt was made to identify plateaus in individual participant data. Instead, correlations were reported between the behaviorally estimated capacity and measures of the gradient of signal strength across changes in set size (e.g., difference in signal between set sizes 2 and 4). (Misleadingly, some authors have changed the *y*-axis label in reproductions of Vogel and Machizawa's (2004) figure from “Amplitude increase from 2 to 4 items” to “CDA asymptote”.) The correlations between the signal gradients and capacity estimates support the claim that the strength of the signals is related to WM ability, but do not provide any evidence for a fixed item limit.

A subsequent study, Xu and Chun (2006), also claimed to find plateaus in fMRI responses, specifically signals in superior IPS that reached a maximum at a set size corresponding to behaviorally estimated capacity. This study tested memory for complex (multipart) as well as simple visual items; capacity estimates were lower for complex items (Alvarez and Cavanagh 2004; Awh, Barton, and Vogel 2007), and the authors claimed that signal plateaus occurred at correspondingly lower set sizes.

Biological responses, for example, of individual neurons to stimulation, cannot become arbitrarily large. It is common therefore to model these responses with saturating nonlinearities, such as the Naka-Rushton equation used to model the contrast response of visual neurons (Albrecht and Hamilton 1982), in which activity asymptotes at a particular maximum level. Critically, for a saturating function this asymptotic value is not attained for any finite input. Instead, as the input becomes large, each increase in input produces a smaller increment in response, such that the response approaches the maximum ever more closely without reaching it. Activities of populations of neurons at the scales probed by fMRI or EEG are expected to behave in the same way, and in the case of fMRI the transfer functions between neural activation and blood oxygenation and between blood oxygenation and MR signal introduce further nonlinearities (Jansen and Rit 1995; Buxton et al. 2004). We therefore hypothesize that an alternative candidate model for describing the relationship between signal strength and set size is a saturation model, in which the signal asymptotes but does not reach a maximum at any set size.

Here we re-examine results of the imaging studies described above. The original analyses looked for abrupt changes in the mean response function relating neural signal to set size, but in doing so ignored the fact that capacity varies across participants; we test the ability of a plateau model based on the variability in individual capacity estimates to fit the data, and we compare the plateau model to an alternative model in which signal strength saturates with increasing set size.

## Materials and Methods

We examined data from three published imaging studies of visual WM, two measuring blood oxygenation level dependent (BOLD) signal strength in posterior parietal cortex using fMRI (Todd and Marois 2004; Xu and Chun 2006) and one assessing amplitude of a posterior EEG signal (the contralateral delay activity, CDA; Vogel and Machizawa 2004). We contrasted two different models of the relationship between signal strength and memory load: a plateau model and a saturation model.

### Data

We analyzed group-level BOLD signal response functions from IPS/IOS recorded during a visual WM task, as reported in Todd and Marois (2004, Figure 2). Capacity estimates for the participants in this fMRI experiment were obtained from Todd and Marois (2005), Figure 3b. For Xu and Chun (2006), we focused on BOLD signals recorded from the same Talairach coordinates identified by Todd and Marois, labeled superior IPS by Xu and Chun, Figure 2; we analyzed all data from experiments matching Todd and Marois’ protocol, that is, simultaneous peripheral presentation; mean and variability of capacity estimates were reported in the same paper (we used the capacity estimate at maximum set size, i.e., 6 items). From Vogel and Machizawa (2004) we analyzed the CDA response function obtained by combining Exps 2–4, as in the paper, Figure 3a; individual capacity estimates were reported in the same paper, Figure 3b (We note that data from only 30 out of 36 participants were presented in this figure; the remaining 6 subjects were excluded from analysis due to excessive eye movements or blinks; Edward Vogel, personal communication). In each case, we extracted means and variances (calculated from reported standard errors [SE]) of signal amplitude at each set size for fitting with the models.

### Plateau Model

**Figure 1.**
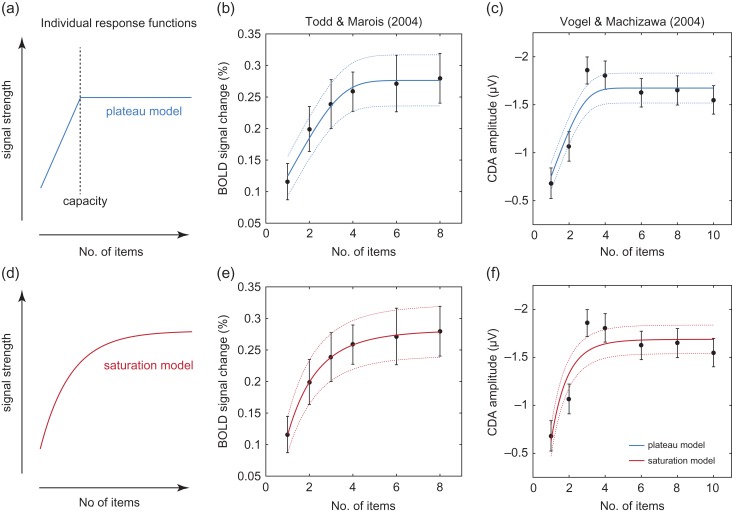
Models and fits. (*a*) Example of an individual response function relating set size to signal strength under the plateau model. According to the plateau model, signal strength increases until the set size matches the individual’s capacity: further increases in set size do not affect the signal strength. (*b*) Black data points indicate group mean data from an fMRI signal in IPS/IOS (Todd and Marois 2004). Errorbars indicate ±1 SE. Solid lines show fits of the plateau model to group means, and dotted lines show SEs predicted by the model. Note that the plateau model predicts a smooth transition to plateau due to averaging across participants with different capacities. (*c*) Black data points indicate group mean data from a recording of the CDA (Vogel and Machizawa 2004), blue lines show fit of the plateau model. (*d*) Individual response function under the saturation model. According to this model, signal strength increases continuously, but each increment in set size has less effect than the one before: signal strength does not reach a maximum at any set size. (*e*, *f*) Data as in (*b*, *c*), red lines show fit of the saturation model.

According to the plateau model, signal strength increases with set size until an individual’s WM capacity is reached; the signal then plateaus and is unchanged by further increases in set size (Fig. 1*a*). Formally, each individual’s signal strength, y, is determined by set size, x, according to a bilinear function:

y=α+βmin{x,k}

where, k is the individual’s memory capacity, and α and β are free parameters corresponding to the slope and intercept of the linear rise, respectively.

In order to fit this model to group-level (mean and variance) data, we assumed that the parameters of the model are independently normally distributed across individuals. The mean and standard deviation of the capacity parameter k are taken from the capacity estimates reported in each paper (or, in the case of Todd & Marois 2004, reported in Todd & Marois 2005), which were calculated from WM task performance using the method of Cowan (2001). (In the case of Vogel and Machizawa (2004), who used a whole-display probe design, Pashler’s (1988) formula would actually have been the more correct calculation; Rouder et al. 2011). This results in a model of group-level data with 4 free parameters, $\{\mu_{\alpha },\mu_{\beta },\sigma_{\alpha },\sigma_{\beta }\}$, corresponding to the group means and standard deviations of parameters α and β (see Appendix for mathematical details).

### Saturation Model

Under the saturation model, signal strength increases with set size according to an exponential function, such that each successive increase in number of items makes a smaller increment to the signal, but the signal does not reach a maximum at any set size (Fig. 1*d*). Formally,

y=α+β(1−exp(−γx)).

We again assumed parameters are independently normally distributed across individuals, resulting in a model of group-level data with 6 free parameters, $\{\mu_{\alpha },\mu_{\beta },\mu_{\gamma },\sigma_{\alpha },\sigma_{\beta },\sigma_{\gamma }\}$, corresponding to the group means and standard deviations of α, β, and γ (see Appendix).

### Model Fitting and Comparison

Models were fit by minimizing the squared error between the model predictions and the data, using the Nelder-Mead simplex method (fminsearch in MATLAB), with multiple starting parameters to avoid local minima. This least-squares fitting procedure is identical to maximum likelihood fitting with a normal error term; models were compared using the Bayesian information criterion (BIC).

## Results

We compared the ability of two models to capture the relationship between set size and signal strength observed in imaging studies of visual WM. According to the plateau model (Fig. 1*a*), signal increases with set size until an individual’s capacity is reached then plateaus. According to the saturation model (Fig. 1*d*), signal increases continuously, following an exponential function. Critically, we assumed that the parameters of the models would vary across individuals, according to a normal distribution.

Data from Todd and Marois (2004) are shown as black symbols in Figure 1*b*,*e*. Todd and Marois interpreted these data as indicating a plateau at 4 items, yet at first glance they appear incompatible with a plateau model, as there is no sharp discontinuity that would indicate a transition from an increasing function to a steady state. However, this would be to overlook the fact that capacity, and hence the point of plateau, is thought to vary from individual to individual; the result is that group mean data will tend to display a curve with a smooth “shoulder.” Based on the distribution of capacity estimates reported by Todd and Marois for their participants, we can predict the expected curvature and fit a model to the group data consistent with our knowledge of individual capacities; this fit is shown by the blue lines in Figure 1*b* (mean ± SE).

The fit of our alternative model, which assumes each individual’s response follows a saturation curve with no point of plateau, is plotted as the red lines in Figure 1*e*. Both plateau and saturation models provide a good account of the data, with the saturation model providing a better fit (ΔBIC = 11.1).

Black symbols in Figure 1*c*,*f* show results of Vogel and Machizawa (2004). These data show a sharp discontinuity that appears, and was interpreted by the original authors, to provide dramatic evidence of a plateau at 3 items. However, this interpretation again fails to take into account the variability in capacity across individuals. As shown by the blue lines in Figure 1*c*, a plateau model based on the reported capacities of the participants predicts a gentler curve, and actually provides a weak fit to the data. The saturation model (red lines in Fig. 1*f*) provides a similar but slightly worse fit (ΔBIC = –3.84).

**Figure 2.**
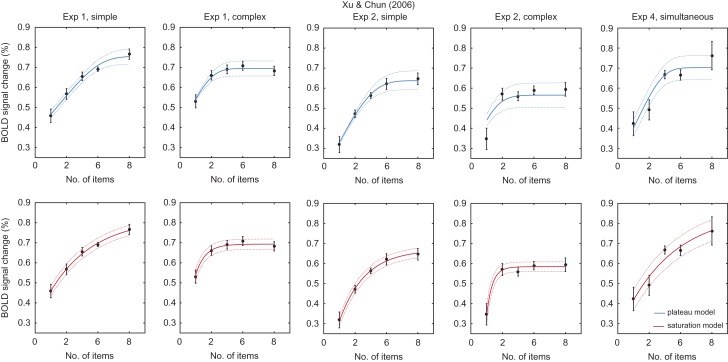
Fits to data from Xu & Chun (2006). Black data points show group-level fMRI response functions recorded from the same posterior parietal coordinates identified by Todd & Marois (2004). Errorbars indicate ±1 SE. Solid lines indicate fits of the plateau model (top, blue) and saturation model (bottom, red) to group means, dotted lines indicate SEs predicted by the models. Panel titles indicate the experiment number and whether memory stimuli were simple or complex; for Exp 4, we examined only data from the simultaneous presentation condition (matching the other studies).

Xu and Chun (2006) followed up Todd and Marois’ work, examining the effect of object complexity on capacity estimates and the fMRI response function. Panels in Figure 2 show results from two experiments contrasting memory for simple and complex stimuli, and an additional experiment with simple stimuli. The saturation model (bottom, red lines) provides a better fit to the data than the plateau model (top, blue lines) in all cases (ΔBIC: Exp 1, simple, 9.69; complex, 1.83; Exp 2, simple, 9.08; complex, 27.3; Exp 4, 2.27). In most instances both models make similar predictions and both provide an acceptable fit to data; the poor fit of the plateau model to data from Exp 2, complex condition, is due to the very low estimated capacity in that condition (mean: 1.33 items).

Collating evidence across the three studies, the saturation model provided a better fit to the data than the plateau model (ΔBIC = 57.4).

## Discussion

Todd and Marois (2004) claimed that BOLD activation in posterior parietal cortex increased with memory load then reached a plateau at a point corresponding to the estimated WM capacity of their participants. We found that this account corresponded quite well with their data, but that the results were better described by a saturation model that did not reach a maximum at any set size.

Vogel and Machizawa (2004) also claimed to observe a plateau corresponding to their participants’ capacity limits, this time in a posterior EEG signal. We found that the sharp discontinuity in slope in their group-level data was in fact inconsistent with the spread of capacities observed for their participants, which predicted a smoother “shoulder” to the curve. Plateau and saturation models made very similar predictions for this data, neither providing a satisfactory fit.

Xu and Chun (2006) reported plateaus in a posterior parietal BOLD signal that tracked the capacities of their participants for simple and complex memoranda. This model provided an acceptable fit to their data in most cases, but the saturation model fit better in every case.

Overall, we conclude that the evidence for plateaus in neural signal in these studies is weak, with a saturation account providing a viable and biophysically plausible alternative. Importantly, we do not dispute that these neural signals are related to information storage; they clearly are influenced by the number of items in the memory array and also the complexity of those items (Xu and Chun 2006). However, because the changes in signal with set size are well described by a continuously increasing function, they do not provide evidence for a fixed upper limit on the number of items stored.

Our analysis was based on fitting plateau and saturation models to group-level statistics (means and standard deviations) obtained from each study. Our ability to differentiate the models may have been enhanced had we had access to trial-by-trial data from individual participants, however, we see no reason to think the outcome would have been different, and the present analysis was sufficient to obtain a very respectable overall BIC difference (>50) favoring the saturation model.

Todd and Marois (2004) argued that their results could not be accounted for by haemodynamic saturation of the neurovascular system, on the grounds that BOLD responses with greater amplitude were possible: this was demonstrated in a control study with a shorter intertrial interval. However, our proposal is that neuronal activity, rather than blood oxygenation, is saturating in these studies. Summation of overlapping BOLD responses would account for the stronger responses in the control study, without requiring a stronger neural response.

In order to explain the behaviorally observed decline in memory precision with set size, resource models propose that the capacity of WM is used to its full extent whenever items are held in memory: the need to divide up this resource when multiple items are stored is the basis for the increase in recall variability (Bays and Husain 2008; van den Berg et al. 2012; Bays 2014; Ma, Husain, and Bays 2014). Similarly, current versions of the item limit model propose that all memory slots are allocated to stimuli regardless of how many items are presented (Zhang and Luck 2008). So no current model directly predicts the existence of a load-dependent neural signal. One possibility is that BOLD and EEG signals, which are primarily driven by synaptic conductances (Logothetis 2008; Buzsáki, Anastassiou, and Koch 2012), are in this case reflecting an overall increase in synaptic processing with set size, rather than an increase in action potentials (Ma, Husain, and Bays 2014). The imaging results would therefore be consistent with the neural resource model of Bays (2014) (see also Schneegans and Bays 2017), in which total spiking activity is held constant (normalized) across changes in memory load.

Another possibility is that load-dependent signals in posterior parietal cortex may only indirectly reflect the storage of visual features taking place elsewhere. This would be consistent with recent findings that stimulus features held in visual WM can be decoded from patterns of BOLD activity in regions that do not have elevated delay period activity, including primary visual cortex (Harrison and Tong 2009; Serences et al. 2009; Riggall and Postle 2012). When multiple items are stored, the fidelity of decoding declines with set size, consistent with a resource account of WM (Emrich et al. 2013; Sprague, Ester, and Serences 2014). In contrast, a number of studies have failed to decode the contents of memory from regions with elevated delay-period activity, including IPS (Emrich et al. 2013; Riggall and Postle 2012; but see Christophel, Hebart, and Haynes 2012; Weber et al. 2016). The sensory recruitment hypothesis proposes that visual working memories are maintained by the same neural architecture in visual cortex that initially processes sensory input. The present results, by demonstrating the paucity of evidence for memory capacity limits in parietal cortex, eliminate one potential objection to this hypothesis.

It should be noted that the proposal that sensory areas are recruited for WM storage is still debated (Serences 2016; Xu 2017), in part because it is unclear how memory representations would evade being overwritten by new sensory input (Bettencourt and Xu 2016). Possibly, resistance to overwriting could be a consequence of proposed dynamical (Emrich et al. 2013; Lundqvist et al. 2016; Sreenivasan et al. 2014) or activity-silent (Rose et al. 2016; Wolff et al. 2017) properties of the memory representation, although the details of such a scheme are yet to be resolved.

If memories are stored elsewhere, why would the BOLD signal in IPS be correlated with memory load? One possibility is that load-dependent activity observed in IPS could reflect control signals determining which stimuli are maintained in WM but not themselves storing feature information. In this case, the apparent plateau in BOLD signal in this region would be interpreted as evidence for a fixed upper limit on the number of items that can be selected for storage, and the present results should be viewed as providing evidence against such a limit. Several studies have linked IPS activity to attentional allocation, observing changes in BOLD signal with the number of items visible even in the absence of explicit memory demands (Mitchell and Cusack 2008; Magen et al. 2009). Similar dissociations between signal amplitude and memory requirements have been observed for the CDA (Tsubomi et al. 2013) and its tactile equivalent (Katus and Eimer 2015). The role of attention in allocating limited WM resources and modulating recall precision is now well established (Emrich, Lockhart, and Al-Aidroos 2017). An attentional interpretation of load-related IPS activity has also been put forward to account for an association with variability in recall precision (Weber et al. 2016).

In conclusion, existing studies of memory load-dependent fMRI and EEG signals do not provide support for a fixed upper limit on the number of items stored. While these signals appear to be at least indirectly related to the information contained in WM, the existing evidence suggests that they progressively saturate with increasing memory load, rather than reaching a sharp plateau at an individual capacity limit. These results are consistent with resource models of WM, in which there is no upper limit, and instead memory fidelity degrades with increasing set size until recall is indistinguishable from noise.

## Appendix

Here we provide mathematical details of the models and their derivations.

**Plateau model**

Consider the bilinear function:

y=α+βmin{x,k},

for independent normally distributed random variables α, β, and k,

$$\begin{array}{c} \alpha ∼N(\mu_{\alpha },\sigma_{\alpha })\\ \beta ∼N(\mu_{\beta },\sigma_{\beta })\\ k∼N(\mu_{k},\sigma_{k}). \end{array}$$

The expected value (mean) of y is given by the following equation:

$$\begin{array}{cc} E[y] & =E[\alpha +\beta \min\{x,k\}]\\ & =E[\alpha ]+E[\beta ]E[\min\{x,k\}]\\ & =\mu_{\alpha }+\mu_{\beta }E[\min\{x,k\}], \end{array}$$

and the variance of y is given by by the following equation:

$$\begin{array}{c} \mathrm{Var}[y]=\mathrm{Var}[\alpha +\beta \min\{x,k\}]\\ \;=\mathrm{Var}[\alpha ]+\mathrm{Var}[\beta ]\mathrm{Var}[\min\{x,k\}]+\mathrm{Var}[\beta ](E[\min\{x,k\}]{)}^{2}\\ \;+(E[\beta ]{)}^{2}\mathrm{Var}[\min\{x,k\}]\\ \;=\sigma_{\alpha }^{2}+\sigma_{\beta }^{2}\mathrm{Var}[\min\{x,k\}]+\sigma_{\beta }^{2}(E[\min\{x,k\}]{)}^{2}\\ \;+\;\mu_{\beta }^{2}\mathrm{Var}[\min\{x,k\}], \end{array}$$

where,

$$\begin{array}{cc} E[\min\{x,k\}] & =\int_{-\infty }^{\infty }p(k\prime )\min\{x,k\prime \}\mathrm{dk}\prime \\ & =\int_{-\infty }^{x}\phi_{\mu_{k},\sigma_{k}}(k\prime )k\prime \mathrm{dk}\prime +\int_{x}^{\infty }\phi_{\mu_{k},\sigma_{k}}(k\prime )x\mathrm{dk}\prime \\ & =\int_{-\infty }^{x}\phi_{\mu_{k},\sigma_{k}}(k\prime )k\prime \mathrm{dk}\prime +x\left(1-\Phi_{\mu_{k},\sigma_{k}}(x)\right)\\ & =\mu_{k}\Phi_{\mu_{k},\sigma_{k}}(x)-\sigma_{k}^{2}\phi_{\mu_{k},\sigma_{k}}(x)+x\left(1-\Phi_{\mu_{k},\sigma_{k}}(x)\right),\\ \mathrm{Var}[\min\{x,k\}] & =E[\min\{x,k{\}}^{2}]-(E[\min\{x,k\}]{)}^{2}\\ & =\int_{-\infty }^{x}\phi_{\mu_{k},\sigma_{k}}(k\prime )k\prime^{2}\mathrm{dk}\prime +\int_{x}^{\infty }\phi_{\mu_{k},\sigma_{k}}(k\prime )x^{2}\mathrm{dk}\prime \\ & \;\;-\;(E[\min\{x,k\}]{)}^{2}\\ & =\int_{-\infty }^{x}\phi_{\mu_{k},\sigma_{k}}(k\prime )k\prime^{2}\mathrm{dk}\prime +x^{2}\left(1-\Phi_{\mu_{k},\sigma_{k}}(x)\right)\\ & \;\;-\;(E[\min\{x,k\}]{)}^{2}\\ & =(\mu_{k}^{2}+\sigma_{k}^{2})\Phi_{\mu_{k},\sigma_{k}}(x)-(x+\mu_{k})\sigma_{k}^{2}\phi_{\mu_{k},\sigma_{k}}(x)\\ & \;\;+\;x^{2}(1-\Phi_{\mu_{k},\sigma_{k}}(x))-(E[\min\{x,k\}]{)}^{2}. \end{array}$$

We fit the model described by these equations to group-level (mean and variance) data as described in the main text, using values of $\mu_{k}$ and $\sigma_{k}$ obtained from reported capacity estimates.

**Saturation model**

Consider the exponential function:

y=α+β(1−exp(−γx))

for independent normally distributed random variables α, β, and γ,

$$\begin{array}{c} \alpha ∼N(\mu_{\alpha },\sigma_{\alpha })\\ \beta ∼N(\mu_{\beta },\sigma_{\beta })\\ \gamma ∼N(\mu_{\gamma },\sigma_{\gamma }). \end{array}$$

The expected value (mean) of y is given by the following equation:

$$\begin{array}{cc} E[y] & \;=E[\alpha +\beta (1-\exp(-\gamma x))]\\ & \;=\;E[\alpha ]+E[\beta ](1\;-\;E[\exp(-\gamma x)])\\ & \;=\;\mu_{\alpha }+\mu_{\beta }(1\;-\;E[\exp(-\gamma x)]), \end{array}$$

and the variance of y is given by the following equation:

$$\begin{array}{cc} \mathrm{Var}[y] & \;=\;\mathrm{Var}[\alpha +\beta (1-\exp(-\gamma x))]\\ & \;=\;\mathrm{Var}[\alpha ]+\mathrm{Var}[\beta ]\mathrm{Var}[\exp(-\gamma x)]+\mathrm{Var}[\beta ](1-E[\exp(-\gamma x)]{)}^{2}\\ & \;\;+(E[\beta ]{)}^{2}\mathrm{Var}[\exp(-\gamma x)]\\ & \;=\;\sigma_{\alpha }^{2}+\sigma_{\beta }^{2}\mathrm{Var}[\exp(-\gamma x)]+\sigma_{\beta }^{2}(1-E[\exp(-\gamma x)]{)}^{2}\\ & \;+\;\mu_{\beta }^{2}\mathrm{Var}[\exp(-\gamma x)], \end{array}$$

where,

$$\begin{array}{cc} E[\exp(-\gamma x)] & \;=\int_{-\infty }^{\infty }\phi_{\mu_{\gamma },\sigma_{\gamma }}(\gamma \prime )\exp(-\gamma \prime x)d\gamma \prime \\ & \;=\;\exp\left(\frac{1}{2}\sigma_{\gamma }^{2}x^{2}-\mu_{\gamma }x\right),\\ \mathrm{Var}[\exp(-\gamma x)] & \;=E[\exp(-\gamma x{)}^{2}]-(E[\exp(\gamma x)]{)}^{2}\\ & \;=\int_{-\infty }^{\infty }\phi_{\mu_{\gamma },\sigma_{\gamma }}(\gamma \prime )\exp(-2\gamma \prime x)d\gamma \prime -(E[\exp(\gamma x)]{)}^{2}\\ & \;=\exp\left(2\sigma_{\gamma }^{2}x^{2}-2\mu_{\gamma }x\right)-(E[\exp(\gamma x)]{)}^{2}. \end{array}$$

We fit the model described by these equations to group-level (mean and variance) data as described in the main text.
